# COVID-19 prevalence and mortality is associated with the allele frequency of CCR5-Δ32

**DOI:** 10.3325/cmj.2021.62.303

**Published:** 2021-06

**Authors:** Mostafa Saadat

To the Editor: I read with great interest the article *Does the CCR5-Δ32 mutation explain the variable coronavirus-2019 pandemic statistics in Europe?* by Starčević Čizmarević et al (1). The authors found no significant association between COVID-19 prevalence/mortality and the CCR5-Δ32 allele frequency in 39 European countries. As mentioned by the authors, European countries share a relatively similar genetic background. Although the prevalence and mortality of COVID-19 differ across European countries, these epidemiologic parameters vary even more between European and non-European countries. The CCR5-Δ32 frequency in European populations is higher than in Asian, especially East-Asian, populations. To determine whether COVID-19 prevalence/mortality follows the geographical distribution of CCR5-Δ32 worldwide, I extended the analysis performed by Starčević Čizmarević et al to 82 world countries.

COVID-19 prevalence/mortality and the number of performed diagnostic tests (per 10^6^ people, as of end of December, 2020) were obtained from the Worldometer website (*www.worldometers.info/coronavirus/countries).* The CCR5-Δ32 frequency was obtained from a previously published article (2). The Human Development Index (HDI) value, reflecting three major dimensions of human development: life expectancy at birth, education, and the gross national income per capita, was used as a potential confounder. Another potential confounder was the number of performed diagnostic tests (Supplementary Table 1(Supplementary Table 1)). While HDI showed normal distribution, COVID-19 prevalence/mortality, the number of preformed diagnostic tests, and CCR5-Δ32 frequency deviated from the normal distribution and were square root-transformed (SR-transformed).

SR-prevalence (r = 0.516, df = 80, *P* < 0.001) and SR-mortality (r = 0.456, df = 80, *P* < 0.001) were significantly associated with the SR-CCR5-Δ32 frequency. To account for the effect of confounding socio-economic factors on COVID-19 prevalence/mortality, multivariable linear regression analysis was used. Table 1 shows the final multivariable models constructed using a backward elimination procedure. In the model, SR-prevalence was significantly positively associated with SR-CCR5-Δ32 frequency (partial r = 0.265, *P* = 0.017). SR-transformed mortality was positively associated with CCR5-Δ32 frequency (partial r = 0.216), but the difference did not reach significance (*P* = 0.053). This means that countries with a high frequency of CCR5-Δ32 allele had a higher COVID-19 prevalence and mortality than countries with a low CCR5-Δ32 frequency. The current findings reveal that the frequency of CCR5-Δ32 mutation can partially explain the difference in COVID-19 prevalence/mortality between populations.

**Table 1:** Multivariable linear regression analysis of the associations between COVID-19 prevalence and mortality and the allelic frequency of CCR5-Δ32 in the 82 countries worldwide*^†^

Variables	Unstandardized coefficients		Standardized coefficients beta	Partial correlations	t	P
	B	StandardError				
**SR-prevalence as dependent variable**						
**Constant**	19.48	17.32	-	-	1.12	0.264
**SR-performed frequency of CCR5-Δ32**	19.69	8.07	0.237	0.265	2.44	0.017
**SR-performed tests**	0.146	0.026	0.551	0.539	5.68	<0.001
**SR-mortality as dependent variable**						
**Constant**	-16.38	6.75	-	-	-2.42	0.018
**SR-performed frequency of the CCR5-Δ32**	2.95	1.50	0.235	0.216	1.96	0.053
**Human Development Index**	35.52	10.68	0.397	0.350	3.32	0.001

*SR – square-root transformed.

**†**The first model was significant with F = 41.49; df = 2, 79; *P* < 0.001; adjusted R^2^ = 0.500. The second model was significant with F = 19.66; df = 2, 79; *P* < 0.001; adjusted R^2^ = 0.315.
