# Supplementary material for: COVID-19 prevalence and mortality is associated with the allele frequency of CCR5-Δ32
Source: Croat Med J. 2021 Jun;62(3):303–4. doi: 10.3325/cmj.2021.62.303 (PMC8275944; doi:10.3325/cmj.2021.62.303)
Supplement: Supplementary Table 1 [file CroatMedJ_62_s002.pdf]

Table S1: Prevalence and mortality of COVID-19 in 82 countries, the allelic frequency of the CCR5-Δ32 and other variables used in the study

Country	Frequency of CCR5-Δ32 (%)	Prevalence (per 10^6 population)	Mortality (per 10^6 population)	Number of diagnostic tests performed (per 10^6 population)	Human Development Index (HDI)
Afghanistan	3.86	1334	56.0	5090	0.511
Albania	5.47	20275	411.0	89062	0.795
Argentina	9.40	35801	952.0	105970	0.845
Armenia	3.29	53745	952.0	200234	0.776
Austria	9.74	39947	689.0	422070	0.922
Azerbaijan	3.95	21472	259.0	215555	0.756
Bangladesh	15.20	3103	46.0	19500	0.632
Belarus	10.22	20564	151.0	422156	0.823
Belgium	9.92	55466	1674.0	594130	0.931
Bosnia and Herzegovi	8.09	33936	1238.0	156535	0.780
Brazil	5.44	35983	914.0	134070	0.765
Bulgaria	7.05	29221	1095.0	166904	0.816
Cameroon	.70	978	17.0	5544	0.563
Canada	7.02	15337	412.0	363376	0.929
Chile	12.02	31719	865.0	336033	0.851
China	.47	60	3.0	111163	0.761
Colombia	5.06	32113	845.0	159013	0.767
Congo, DR	2.50	1272	19.0	13027	0.480
Croatia	7.52	51519	958.0	249025	0.851
Cuba	7.07	1048	13.0	130412	0.783
Czech Re	10.68	67054	1087.0	352869	0.900
Denmark	12.32	28174	224.0	1821172	0.940
Dominican Republic	4.27	15665	221.0	79785	0.756
Ecuador	3.61	11954	789.0	42113	0.759
Egypt	2.93	1337	74.0	9681	0.707
El Salvador	9.05	7068	205.0	95594	0.673
Eritrea	.26	370	0.8	6635	0.459
Estonia	15.63	21092	173.0	477291	0.892
Ethiopia	.00	1068	17.0	15469	0.485
Finland	12.93	6512	101.0	444806	0.938
France	10.63	40100	989.0	535994	0.901
Georgia	4.24	57065	629.0	470744	0.812
Germany	11.10	20800	407.0	414703	0.947
Ghana	2.81	1745	11.0	21202	0.611
Greece	5.17	13355	465.0	325327	0.888
Hungary	11.19	33428	989.0	275409	0.854
India	2.09	7417	107.0	124060	0.645
Indonesia	3.53	2703	81.0	26748	0.718
Iran	4.02	14493	653.0	89515	0.783
Iraq	3.90	14636	315.0	111811	0.674
Ireland	12.37	18483	451.0	478239	0.955
Israel	10.58	46019	362.0	869479	0.919
Italy	6.27	34877	1227.0	440252	0.892
Japan	4.27	1824	27.0	38422	0.919
Jordan	2.71	28720	374.0	309687	0.729
Kazakhstan	10.28	8191	120.0	277938	0.825

**Kenya**	2.98	1774	31.0	19254	0.601
**Kyrgyzstan**	9.67	12319	206.0	90205	0.697
**Latvia**	15.09	21809	339.0	467601	0.866
**Lebanon**	2.54	26653	216.0	292040	0.744
**Lithuania**	12.07	52012	539.0	601676	0.882
**Luxembourg**	9.22	73846	789.0	2653941	0.916
**Macedonia**	5.17	39998	1201.0	192396	0.774
**Mexico**	6.81	10909	964.0	27751	0.779
**Moldova**	12.05	35942	741.0	138325	0.750
**Montenegro**	11.08	76813	1086.0	288502	0.829
**Morocco**	3.27	11828	199.0	120046	0.686
**Netherlands**	10.33	46460	666.0	336388	0.944
**Nigeria**	4.38	420	6.0	4543	0.539
**Norway**	16.41	9107	80.0	512352	0.957
**Pakistan**	2.86	2151	45.0	30023	0.557
**Peru 194**	4.64	30574	1135.0	166330	0.777
**Philippines**	3.81	4297	84.0	61243	0.718
**Poland**	10.55	34230	755.0	190452	0.880
**Portugal**	6.97	40630	678.0	548976	0.864
**Romania**	8.76	32978	822.0	250227	0.828
**Russia**	10.87	21644	391.0	621027	0.824
**Serbia**	6.99	38755	368.0	263353	0.806
**Slovakia**	9.44	32877	392.0	261676	0.860
**Slovenia**	7.14	58775	1297.0	324385	0.917
**South Africa**	13.16	17713	477.0	110737	0.709
**South Korea**	2.22	1184	18.0	82155	0.916
**Spain**	8.14	41415	1087.0	577712	0.904
**Sri Lank**	.54	2018	10.0	57607	0.782
**Switzerland**	9.41	52069	880.0	409748	0.955
**Thailand**	3.21	98	0.9	17426	0.777
**Tunisia**	3.87	11711	394.0	51829	0.740
**Turkey**	3.40	26047	246.0	288986	0.820
**Ukraine**	9.75	24198	425.0	128110	0.779
**United Kingdom**	11.78	36564	1080.0	806468	0.932
**Venezuela**	4.84	3999	36.0	85033	0.711
**Vietnam**	0.77	15	0.4	14641	0.704
